# Patient Experience Ratings: What Do Breast Surgery Patients Care About?

**DOI:** 10.7759/cureus.28846

**Published:** 2022-09-06

**Authors:** Betty Fan, Folasade Imeokparia, Kandice Ludwig, Lisa Korff, Joanna Hunter-Squires, Bindhupriya Chandrasekaran, Sandeep Samra, Joshua Manghelli, Carla Fisher

**Affiliations:** 1 Surgical Oncology, Indiana University School of Medicine, Indianapolis, USA; 2 Patient Experience, Indiana University Health, Indianapolis, USA

**Keywords:** quality improvement projects, patient’s satisfaction, general surgery and breast cancer, patient reported experience, breast cancer outcomes

## Abstract

Introduction

Patient experience is essential in the overall care; physicians often receive patient reviews evaluating their consultation encounters. Patient experience surveys can be a helpful tool to identify areas to target for improvement. We sought to evaluate what factors influenced breast surgery patients' reviews of their clinic visits.

Methods

Prospective surveys from 2018-2020 were reviewed from a single institution. Surveys were sent to all patients within 48 hours after visiting one of our breast surgery clinics, and patients were asked their preferred mode of contact for the survey. Patients responded to surveys with scores of 0-10, with 0 as "not likely" and 10 "extremely likely" to recommend the provider's office. Scores 0-6 were considered negative, 7-8 neutral, and 9-10 positive. Positive/Negative comments from patients were reviewed and classified according to mention of surgeon, clinic staff/team, clinic processing, and facility amenities.

Results

744 out of 2205 patients contacted responded to the survey, resulting in a 33.7% response rate. Of this cohort, 47.6% (354/744) were new patients, and 52.4% (390/744) were established patients. Interactive voice response (IVR) and email, per patient indicated preferred mode of survey communication, had the highest responses. The average patient score was 9.5. Most ratings were positive (91.3%, 679/744), followed by neutral comments (5.2%, 39/744). There were 3.5% (26/744) which were negative ratings. Of those who responded, 47.7% (355/744) left a comment with their score. Surgeon-specific remarks were often noted in positive comments, followed by clinic staff/team comments. Negative comments most commonly referenced clinic processes.

Conclusion

Patient satisfaction surveys provide a window into creating the best patient experience. Further efforts to address these factors affecting patient experiences should be made to continue improving patient care.

## Introduction

The patient experience describes an individual's experience of illness or injury and how healthcare treats them. Surveys and satisfaction scores to assess the patient experience have become increasingly important factors in evaluating performance in healthcare. Recently, patient experience grading has even been used as a reflection of the future viability of healthcare organizations [1]. Patient experience data can detect essential areas to target for improvement in hospital systems [2]. Several studies indicate that patients with higher satisfaction levels may be more prone to increased adherence to recommended medical therapies, better clinical outcomes, fewer patient safety issues within hospitals, and fewer healthcare resources [3]. A study on inpatient mortalities in acute myocardial infarctions found that higher patient satisfaction was associated with improved guideline adherence and lower inpatient mortality rates [4]. Therefore, patient experience measures, when used correctly, can be appropriate quality measures that complement clinical performance evaluations [3].

Furthermore, patient experience reviews increasingly influence physician performance evaluation [1]. Though initially, it was uncertain if this was an appropriate method to assess physician performance quality, several studies have shown a relationship between improved physician-patient communication and perception to be influential in better clinical results [5-7].

The comprehensive management of breast surgery patients has become increasingly complex. This multi-leveled care process includes tailored discussions of complicated subject matters with multiple providers, some of which may be outside the breast surgeon's influence. There is currently limited information regarding what factors breast surgery patient experiences are most influenced by during their visits. We sought to evaluate what factors affected patient reviews during a breast surgery clinic visit to understand what matters most to this group of patients.

This study was presented as a meeting poster at the 2021 American Society of Breast Surgeons Virtual Meeting on April 29, 2021.

## Materials and methods

Database

A review of prospective breast surgery patient experience surveys collected from January 1, 2018 -December 31, 2020, was conducted from a single healthcare network encompassing three hospital sites and six breast surgeons. This included benign and breast cancer patients. Breast surgeon genders were identified as five females and one male, and breast surgeon race was reported as four Caucasian and two African American. Breast surgeon years in practice ranged from 0-5 years to >20 years. The study was deemed IRB (institutional review board)-exempted by the Indiana University School of Medicine (Protocol #: 11142).

Survey collection

Institutional surveys were sent to all new or established patients within 48 hours following their visit to one of our outpatient breast surgery clinics. Patients could only receive a survey once every seven days from different hospital survey locations and only once every 90 days from the exact hospital location. Patients were asked at their appointment check-in if they preferred email, text (SMS), or an interactive voice response (IVR) telephone call for their survey. Surveys were sent via patient preference mode; two attempts were made for email and SMS and three for IVR.

The list of survey questions is as follows:

1) On a scale of 0-10, where 0 is not likely and 10 is extremely likely, how likely is it that you would recommend this hospital (provider office) to a friend or family member? 

 a) What is the primary reason for your score?

2) Did we spend enough time to discuss what matters most to you?

3) Do we make it easy for you?

Scoring

Scores were recorded from 0-10, with 0 as "not likely" and 10 as "extremely likely" to recommend the provider's office to a friend or family member. Scores 0-6 were considered negative, 7-8 neutral, and 9-10 positive, consistent with the Net Promoter Score (NPS) system [8]. Comments from patients for their given score were reviewed and classified into four categories: surgeon, clinic staff/team (includes nurses, mid-levels, oncology clinic staff), clinic processing (i.e., wait times, check-in process, check-in/front desk personnel), and facility amenities (i.e., ease of parking, location) as indicated. If comments had more than one category mentioned, both were recorded and included as applicable. Comments that were vague or did not easily fall into one of the four categories were excluded from the analysis. 

Statistical analysis

Categorical variables were compared using chi-squared analysis. Descriptive data were presented as numbers and percentages. Statistics were done using SPSS Version 27. A P-value of ≤0.05 was considered statistically significant.

## Results

**Figure 1:**
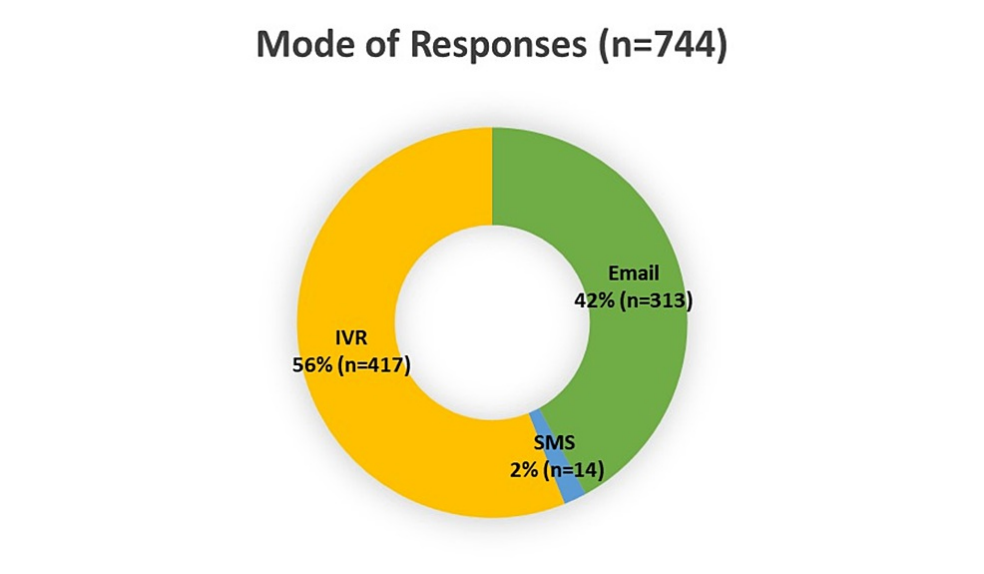
Mode of Responses IVR = interactive voice response (telephone call) SMS= text messaging

**Figure 2:**
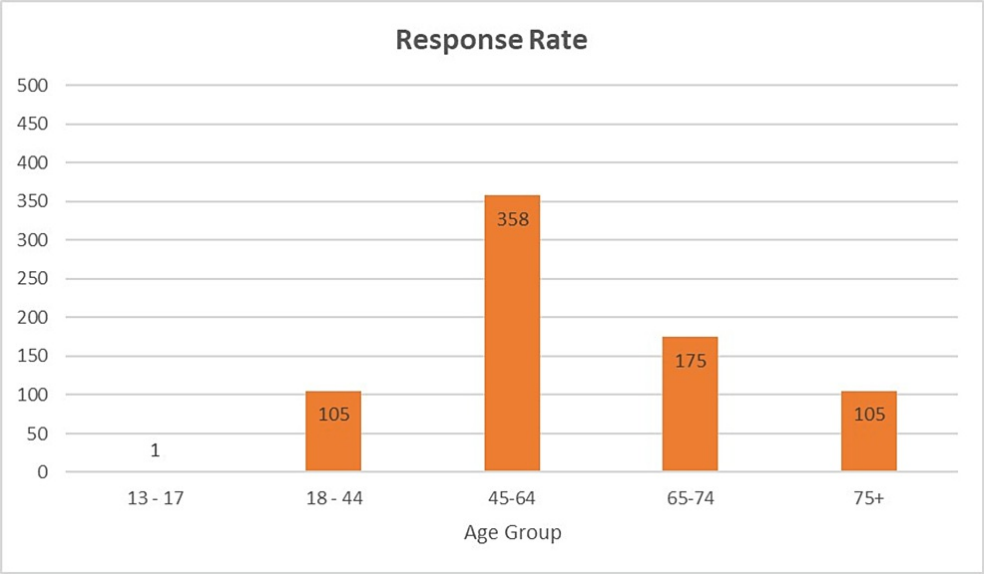
Response Rate by Age Group

**Figure 3:**
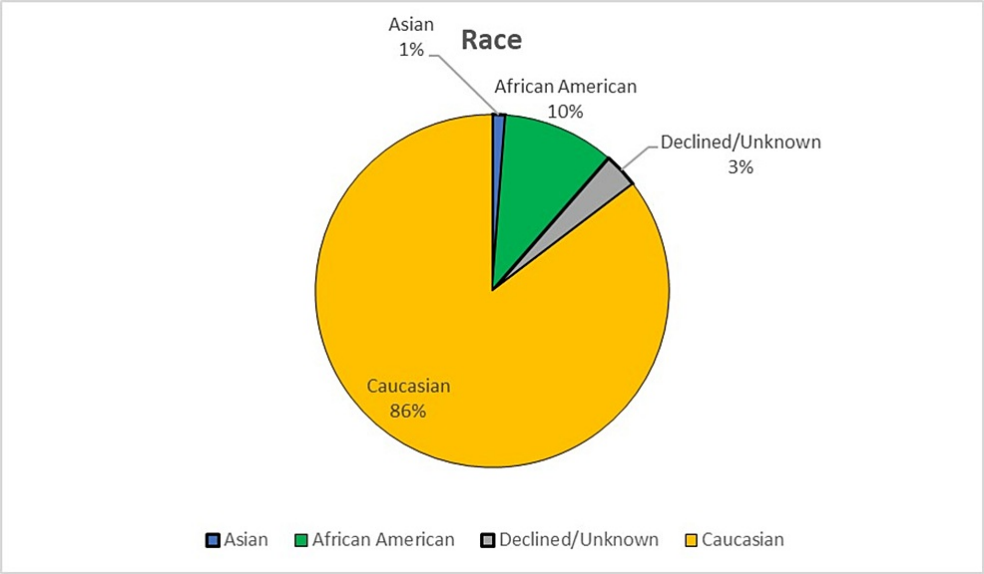
Race of Respondents

**Figure 4:**
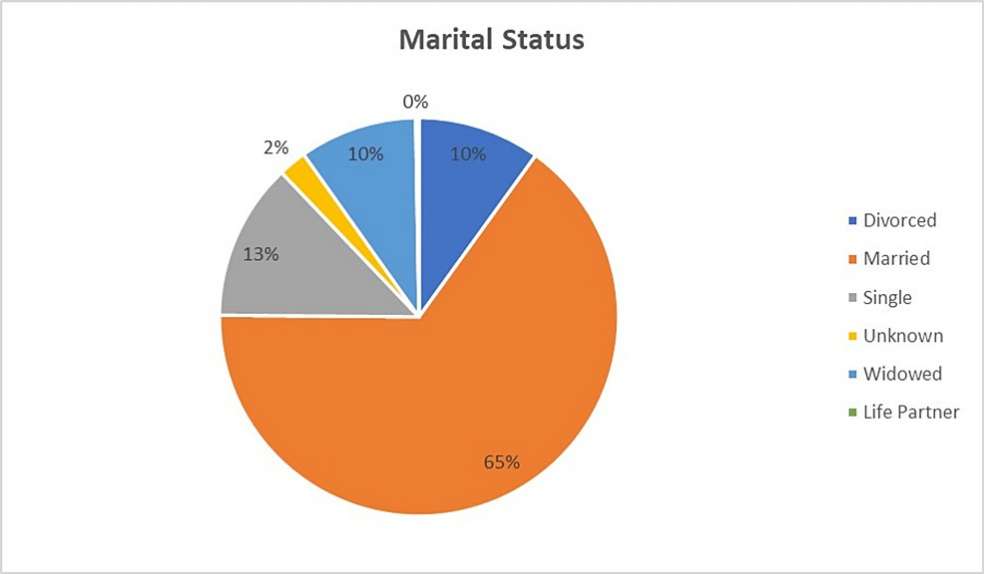
Marital Status of Respondents

Two thousand two hundred five patients were seen and contacted between January 1, 2018, and December 31, 2020, for patient experience surveys. Seven hundred forty-four patients responded and were included in the analysis, resulting in a 33.7% response rate. Of the patients who responded, 47.6% (354/744) were new patients, and 52.4% (390/744) were established patients. IVR and email had the highest number of responses (figure 1). Response rates by age group are shown in figure 2. In the cohort, 99% (740/744) were female, <1% (3/744) were male, and one patient did not disclose gender. Except for one patient, English was listed as the preferred language. Race and marital status information are shown in figures 3, 4.

**Figure 5:**
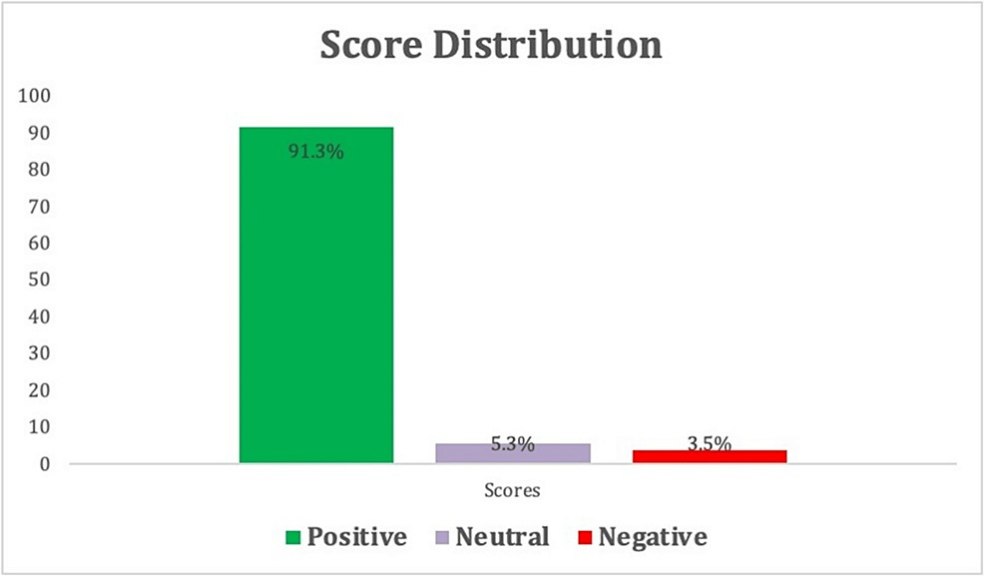
Score Distribution

The average patient experience score was 9.5 (range 0-10). Most ratings were positive (91.3%, 679/744), followed by neutral (5.2%, 39/744). Only 3.5% (26/744) of the ratings were negative (Figure 5).

**Figure 6:**
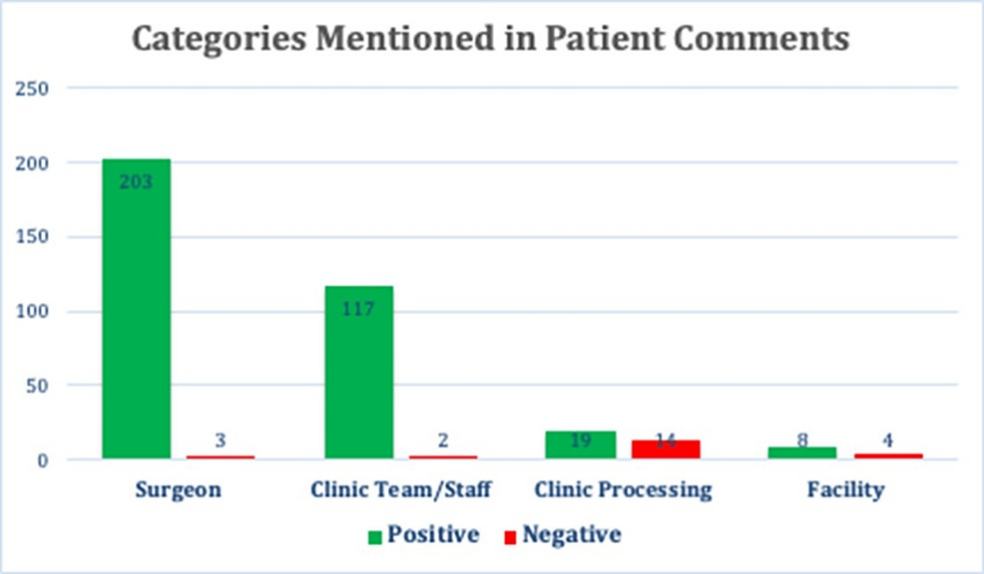
Categories Mentioned in Comment

**Figure 7:**
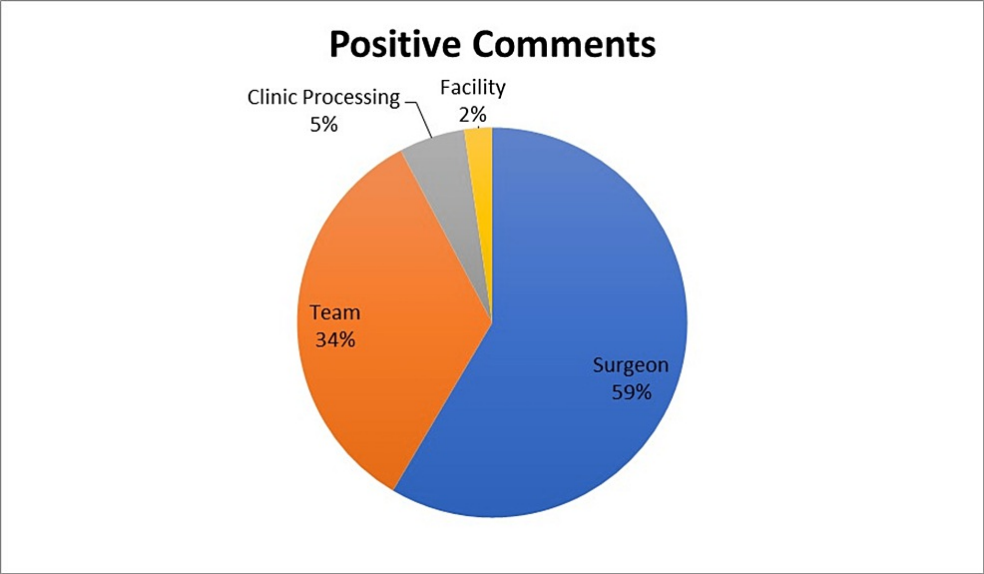
Distribution of Categories by Positive Comments

**Figure 8:**
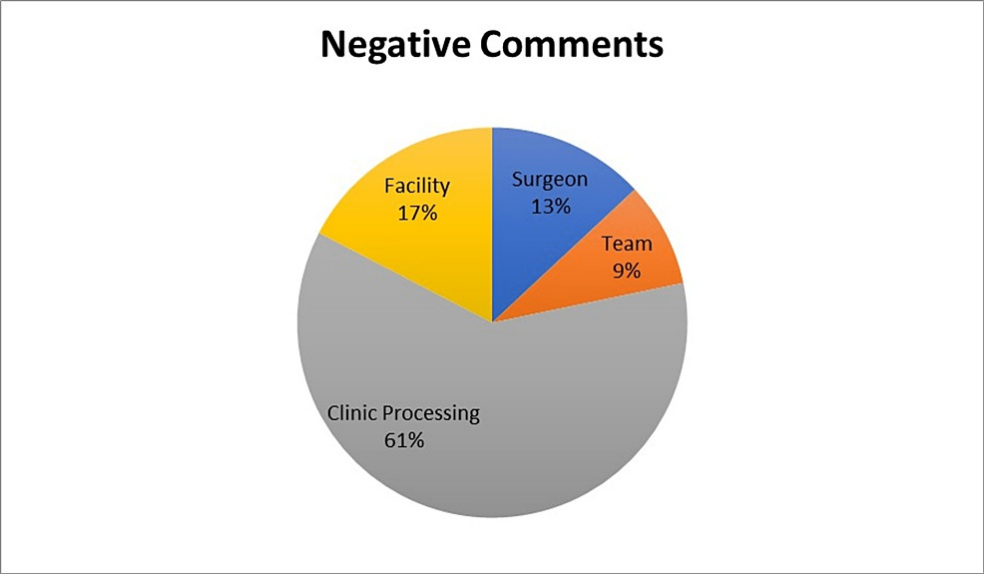
Distribution of Categories by Negative Comments

Of patients who responded to the survey, 47.7% (355/744) left a comment with their score. Examples of patient comments and how they were classified are shown in Table 1. Surgeon-specific remarks were most often noted among the positive comments, followed by mentions of the team or clinic staff. Negative comments were most commonly for clinic processes such as long wait times. Few comments were related to facility amenities such as ease of parking or clinic aesthetics (figure 6-8).

**Table 1:** Examples of Comments and Categorization Process (specific surgeon names were replaced with “***” for anonymity)

	Positive Comments	Negative Comments
Surgeon	“Dr. *** is a skilled surgeon and clearly communicates with her patients.” “Dr. *** was very professional, very friendly, and she answered every question. And she gave her patient time they need. And I'm really happy, very happy, for you know, to be her patient. And I recommend her for all my friends and anybody that I know. Very satisfied. Thank you.”	“Felt like doctor was talking at me not to me.” “… I was not satisfied with the service of the provider that completed my consultation.”
Clinic Team/Staff	“All staff are warm and friendly. Information is presented clearly, first orally and then in writing. Nurse navigators is readily available to answer follow up questions.” “Everyone was very helpful and seemed to care about me not just another number or another dollar. “ “Friendly and professional staff.“	“… the MA when she did my vitals, she did everything incorrect. She was in a hurry, therefore; all my vitals were incorrect.”
Clinic Processing	“… Minimal wait time.” “I got there early--and my appointment started almost as soon as I got there. That is unusual for lots of medical offices!”	“I had to wait a long time to be taken back to consult. That was incredibly weird and long and after the time my appointment was.” “I would have given a higher score, but the wait time was an hour past my appointment time.”
Facility	“Everyone very nice, listened well, office easy to find.” “… everything was clean and the parking is free…” “The office was a calm environment, very friendly and kind…”	“… Also was made to feel most uncomfortable because gown given to me was most likely an extra small … “

Chi-square analysis showed no difference in the likelihood of a negative, neutral, or positive comment from a new or established patient (p=0.291). No further statistical analysis could be performed stratifying positive and negative comments due to the small number of negative comments.

## Discussion

Patient experience surveys of breast surgery patients in our system showed overall high levels of satisfaction with their experience. Surgeon-specific comments were the most common driver for positive experiences contributing to 59% of the positive comments. This is a reassuring finding as the physician-patient relationship continues to contribute to better clinical outcomes. A study by Chen et al. found that the more treatment outcomes discussed by physicians with patients, the higher the patient satisfaction ratings were at baseline and even in follow-up [9]. A similar finding was shown in the study by Ong et al., which confirmed that doctor-patient communication during oncology consultations was related to patients' quality of life and satisfaction [5]. Perhaps in breast surgery, an improved surgeon-patient relationship could theoretically translate into improved compliance and better outcomes. Kahn et al. showed that patient-centered care significantly predictor adherence to long-term tamoxifen use [7].

While physicians should continue to strive to serve patients in a supportive manner, caution should still be mentioned as hyperawareness or overemphasis on patient satisfaction scores as the sole driver of physician performance could also have negative consequences. Overemphasis on patient experience as the main reflection of stellar professional performance could be inappropriate. As demonstrated by Li et al., an unintended consequence of patient satisfaction surveys was altering surgeon clinical practice beyond standard care to meet patient expectations. These actions could include unnecessary referrals, prescribing medications (such as opioids), and ordering additional imaging tests to avoid patient dissatisfaction [10]. Most surgeons reported that these changes did not ultimately result in any clinical changes in outcome or management [10]. Therefore, although patient satisfaction surveys may be a tool to aid in quality performance evaluations, they should still be used judiciously as there may be unintended consequences if healthcare quality measurements are only reflected by patient perception. Additionally, the NPS scoring system may have limitations and not fully encompass a patient's experience, a limitation that applies to many patient experience surveys [8,11,12].

Clinic team interactions were frequently cited as cause for positive experiences, with 34% of positive comments noting their interactions with the clinic staff. This suggests that a breast surgeon's clinical team, such as their oncology nurses and mid-level providers, can influence patients' clinic visit experiences. Attention to selecting support staff who are empathetic and dedicated to the care of breast surgery patients seems to influence patient experiences for breast surgery patients positively.

Very few positive comments referred to the amenities of the hospital or clinic. This likely reflects that patients are focused primarily on the healthcare personnel treating their diseases rather than external factors.

Although occasional negative comments towards the surgeon or clinic staff were found in our review, most negative patient experience evaluations were due to clinic process issues (61%). Long wait times and poor check-in experiences were the most frequently cited comments for negative experiences in our study. Unsurprisingly, patients prefer waiting times to be reasonably short [13]. However, it can often be difficult for breast surgeons to gauge the length of time needed for a new consultation because breast diseases and cancer range in complexity, and patient-driven discussions can be unpredictable. Studies regarding the psychology of patient wait-time experiences note some proactive techniques to mitigate the negative effect of wait times. These can include proactively informing patients of delays, apologizing for delays when they occur, and providing opportunities for diversion for the patient (i.e., magazines, pamphlets, and technology to allow patients to leave and come back when the doctor is ready) [14]. Understanding that wait times may be unavoidable in the care of breast surgery patients, implementing initiatives to ameliorate the negative effects on the patient may improve patient experiences.

Response rates were highest in patients age-45-64, which may correlate with the median age of breast cancer in America. IVR, followed by email, yielded the highest percentage of patient responses. The findings of this study could guide future efforts to improve patient survey response rates.

Using our findings in this study could help institutions and physicians who treat breast cancer target high-yield areas to address to make the most positive impact on patient satisfaction. For instance, given that clinic processes often contribute to negative scores, efforts to minimize patient wait times or offer alternative options when physicians are late may be helpful interventions. 

Limitations of this study include the single-institution database. Patients seen at our facility were mostly Midwestern, and generalizability to other geographic communities may be limited. Additionally, comments were subjective, and attempts at standardizing them into categories could be subject to author interpretation. As with all survey studies, the phrasing of the patient-directed questions may also inadvertently narrow response content.

## Conclusions

Patient satisfaction surveys provide a window into creating the best experience for all patients. Based on our data for breast surgery patients, surgeons are the primary driver behind positive experiences, followed by team and staff interactions. Frustrations with clinic processing, such as long wait times, are the primary reason for dissatisfaction among breast surgery patients. Further efforts to address these factors affecting patient experiences should be made to continue improving patient care. As an emphasis on these scores can affect surgeons' quality metrics, a good understanding of these drivers is essential for healthcare systems.
